# Effect of MLC901 on MIR30C–5P expression, TGF-Β expression, VEGF receptor expression, degree of axon demyelination and changes in neuropathic pain behaviour in experimental animals experiencing neuropathic pain with circumferential spinal stenosis method

**DOI:** 10.1016/j.amsu.2022.104489

**Published:** 2022-08-27

**Authors:** Bambang Priyanto, Andi Asadul Islam, Mochammad Hatta, Agussalim Bukhari, Rohadi Muhammad Rosyidi

**Affiliations:** a Department of Neurosurgery Medical Faculty of Mataram University, West Nusa Tenggara General Hospital, Mataram, Indonesia; b Doctorate Program, Faculty of Medicine, Hasanuddin University, Makassar, Indonesia; c Departement of Neurosurgery, Faculty of Medicine, Hasanuddin University, Makassar, Indonesia; d Molecular Biology and Immunology Laboratory, Faculty of Medicine, Hasanuddin University, Makassar, Indonesia; e Departement of Nutritional Sciences, Faculty of Medicine, Hasanuddin University, Makassar, Indonesia

**Keywords:** Neuropathic pain, Spinal stenosis, Therapy

## Abstract

Neuropathic pain is a major problem whose pathogenesis is not known yet, which makes it difficult to treat. Effective treatment of neuropathic pain usually uses multimodal therapy that takes a long time but causes major health problems, which are commonly found in women over 50 years of age and are generally caused by lumbar radiculopathy due to lumbar spinal stenosis. The narrowing of the spinal canal resembles an ischemic condition that can increase the expression of VEGF in the dorsal root ganglion and then result in shortened walking distance (intermittent claudication). The effect of VEGF is thought to be through binding to VEGFR1 and VEGFR2, whose levels are increased in conditions of hyperalgesia and neuropathic pain. Immune mechanisms play a role in the pathogenesis of neuropathic pain, through the balanced process of pro-inflammatory cytokines and anti-inflammatory cytokines, TGF-β, which are immunosuppressive. MLC901 is a simplified traditional medicine formula from MLC601, which affects the nervous system through three main mechanisms, namely neuroprotection, neuro-regeneration and neuro-restoration. Elevated levels of MLC901 promote angiogenesis. This review discusses the effect of MLC901 on miR30c-5p expression, TGF-β expression, VEGF receptor expression, degree of axon demyelination and changes in neuropathic pain behaviour in experimental animals experiencing neuropathic pain using the circumferential spinal stenosis method. These findings may provide new targets for further scientific research on the molecular mechanisms of neuropathic pain and potential therapeutic interventions.

## Highlights

• Neuropathic pain is a major problem whose pathogenesis is not known yet.
• The narrowing of the spinal canal resembles an ischemic condition that can increase the expression of VEGF in the dorsal root ganglion and then result in shortened walking distance (intermittent claudication).
• This review discusses the effect of MLC901 on miR30c-5p expression, TGF-β expression, VEGF receptor expression, degree of axon demyelination and changes in neuropathic pain behaviour in experimental animals experiencing neuropathic pain using the circumferential spinal stenosis method.

## 1 Introduction

**Fig. 1:**
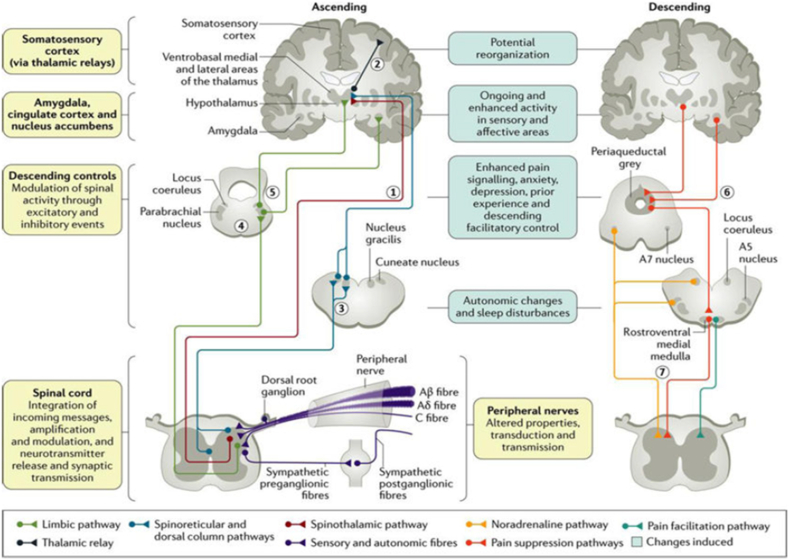
Pain transmission pathway [7].

Neuropathic pain is defined as a pain that is initiated or caused by a primary lesion or dysfunction of the nervous system and may arise as a result of lesions or diseases affecting the somatosensory system. There are molecular changes including the emergence of the c-fos gene, a proto-oncogene involved in regulating cell structural and functional changes, in the spinal cord in response to peripheral stimuli [1,2]. Spinal disorders such as radiculopathy due to disc herniation, spinal stenosis, or spinal cord injury are common causes of neuropathic pain [3]. Several studies reveal the role of spinal neuroimmune responses to pain, namely the discovery of inflammatory mediators such as TNF, IL-1, IL-6, NGF and prostaglandin E2 in inflammatory exudates. Synthesis and release of inflammatory mediators besides functioning in the healing process, also increases pain by lowering the excitation threshold and attracting other nociceptors. Proinflammatory mediators such as NGF, NO, IL-1, IL-6 and TNF will be secreted by the immune system in response to cell damage or injury. Proinflammatory cytokines contribute to axonal damage, as well as modulate spontaneous nociceptor activity and stimulate sensitivity [4]. The simplified MLC901 of MLC601 containing 9 types of herbs has been widely used in China and is starting to be used in Europe, showing the same effectiveness as MLC601 in cellular preclinical studies and stroke in animal models. MLC901 exerts an influence on the nervous system through three main mechanisms, namely neuroprotection, neuro-regeneration and neuro-restoration. Elevated levels of MLC901 promote angiogenesis by modulating the expression of angiogenesis-related factors such as Hif1α, EPO, VEGF and Ang1/Ang2. These factors are known to not only mediate endothelial cell proliferation, but also regulate differentiation, regression, and vascular permeability, which is one of the neuro-regeneration mechanisms after MLC901 administration [5,6] (see Table 1, Fig. 1).

**Table 1:** Summary of models of peripheral nerve neuropathic pain in experimental animals^4^.^5^.

Model	Procedure	Pain-related Behaviour	Clinical Correlation	Onset	Peaks	Duration	PA Histology
Compression of the dorsal root ganglion	Unilateral stainless implantation at foramen L4 or dorsal root ganglion L5	Asymmetrical gait and posture, mechanical allodynia, mechanical hyperalgecia and heat spontaneous pain	Chronic low back pain, sciatica, hyperalgecia, mechanical allodynia	1 days	1–2 days: mechanical allodynia and heat hyperalgecia 4 days: mechanical hyperalgecia 7 days: cold hyperalgecia <1 weeks: spontaneous pain	2 weeks: mechanical allodynia 3 weeks: heat hyperalgecia 5 weeks: spontaneous pain	
Spinal Nerve Ligation	Strong binding to unilateral L5-6 segment (silk 6.0)	Asymmetrical gait and posture, mechanical allodynia and cold, heat hyperalgecia, spontaneous pain, lost with sympathectomy	Causalgia pain	1 days (allodynia) 3 days (hyperalgecia)	1–2 weeks	5 weeks	Sympathetic sprouting to the dorsal root ganglion
Partial Nerve Ligation	Strong binding on 1/3 – ½ unilateral ischiadicus nerve (silk 8.0)	Asymmetrical gait and posture, mechanical allodynia, heat and mechanical hyperalgecia, spontaneous pain, may lost with sympathectomy	Causalgia pain on the contralateral side, sympathetically influenced pain	In hours	Depending on the behavioural test and the parameters being assessed	>7 months	N/A
Spared Nerve Injury	Ligation (silk 5.0) and transection of the distal tibial and peroneal nerves	Asymmetrical gait and posture, mechanical allodynia and cold on the lateral hind leg, mechanical hyperalgecia and heat on the lateral hind leg	Group of neuropathic pain symptom	<24 h	14 days	>6 months	Sympathetic sprouting to the dorsal root ganglion on L4
Chronic Constriction Injury	Loose binding on ischiadicus nerve	Asymmetrical gait and posture, mechanical allodynia and cold, heat hyperalgecia and autotomy chemical	Severe pain on peripheral mononeuropathy, like spontaneous pain, dysesthesia complex regional pain syndrome (causalgia, reflex sympathetic dystrophy)	2 days	10–14 days	2 months	No damage to the proximal axon of the lesion, selective loss of large myelinated nerve fibers, muscle atrophy, ipsil and contralateral lamina I-II transsynaptic degeneration, axon sprouting to the DRG
Cuff neuropathy	Placing a fixed diameter polyethylene cuff on the ichiadicus nerve	Asymmetrical gait and posture, mechanical allodynia and cold, autotomy	Severe pain on peripheral mononeuropathy, like spontaneous pain, dysesthesia	3 days	10–14 days	28 days	Permanent loss of large myelinated axons distal to the lesion, Wallerian concomitant degeneration, inflammatory reaction, increase of sympathetic axon
Axotomy	Unilateral ichiadicus nerve cutting	Asymmetrical gait and posture, Autotomy, may lost with sympathectomy	dolorosa anesthesia	2 weeks	107 days	N/A	Neuroma, distal nerve degeneration, axon sprouting to the DRG
Nerve Destruction	Ichiadicus nerve destruction using serrated forceps	mechanical hyperalgecia and temperature	N/A	3 weeks	N/A	52 weeks	Wallerian degeneration
Cauda Equina compression	Provide compression media	Motoric disorders, hyperalgecia, claudication	Symptoms of nerve compression, Neurogenic intermittent claudication	1 days	14 days	N/A	Axon demyelination

## 2 Neuropathic pain

Neuropathic pain is pain that occurs because of a primary lesion or dysfunction of the nervous system and can arise from lesions or diseases that affect the somatosensory system. Neuropathic pain is non-self-limiting and the pain experienced is not biologically protective but it occurs within the pathological process of the disease itself. This pain is triggered by the presence of neurotransmitters in response to stimulation of polymodal A-delta and C fibre receptors located in the skin, bone, connective tissue, muscle and visceral organs. These stimuli can be mechanical, chemical and temperature, as well as infections and tumours. This stimulus reaction results in the secretion of neurotransmitters such as prostaglandins, histamine, serotonin,

Substance P, as well as somatostatin (SS), cholecystokinin (CCK), vasoactive intestinal peptide (VIP), calcitonin gene-related peptide (CGRP) and so on [8]. Diseases that can cause neuropathic pain include diabetic neuropathy, post-herpetic neuralgia, trigeminal neuralgia, and spinal cord injury pain due to spinal narrowing [1].

Peripheral and central neuropathic pain originates from neuron sensitization as a noxious stimulus through the pain pathway to the centre. Part of this pathway starts from the dorsal horn, spinothalamic tract (somatic structure) and dorsal column (for visceral), to the sensory thalamus, limbic, prefrontal cortex and insular cortex [8].

The characteristics of neuronal sensitization depend on the increase in neuronal activity, the low threshold of the stimulus for the activity of the neuron itself, and the wide spread of the receptor-containing area which results in increased bursts of various neurons [8]. This sensitization is generally associated with denervation of nerve tissue due to the lesion coupled with continuous stimulation, the presence of afferent impulses originating from peripheral and central and also depends on the activation of ion channels in the axon associated with AMPA/kainat and NMDA receptors [9].

Symptoms of neuropathic pain can be positive symptoms, paraesthesia, dysesthesia, hyperalgesia, and allodynia. Negative symptoms in the form of sensory abnormalities (hypoesthesia and hypoalgesia), weakness and motor abnormalities, changes in reflexes. Patients may complain of spontaneous pain, burning and intermittent sharp electric shocks. While secondary pathological changes such as spasticity, micturition and defecation disorders and autonomic dysfunction are responsible for different pain conditions [10].

The problem with neuropathic pain is therapy related to neuronal damage and is irreversible. This generally occurs due to the apoptotic process that is triggered either through intrinsic modulation of calcium in the neurons themselves or as a result of the inflammatory process as an extrinsic factor. This incident underlies the concept of chronic irreversible pain in the nervous system. On this basis, neuropathic pain should be treated as quickly as possible to avoid the process leading to plasticity as chronic pain [8].

### 2.1 Peripheral mechanism

Many peripheral factors play a role in neuropathic pain conditions, including abnormal nociceptor sensitization, the emergence of abnormal adrenergic sensitivity in nociceptors, the ectopic activity formation in damaged nerve cells or in the dorsal root ganglion, the formation of ephaptic connections in demyelinated axons, and ion gate disturbances [11].

On observation after nerve injury, there was an increase in the number of spontaneous activation of the injured afferent fibres, known as ectopic discharge. This spontaneous activation was initially thought to be associated with a neuroma but is now known to also originate in the dorsal root ganglion and at several points along the nerve. This spontaneous activation is thought to be caused by an increase in membrane oscillations so that they reach the activation threshold more quickly. This activation is further amplified due to cross-stimulation of surrounding neurons [11].

Other observations have shown that after nerve injury there is a change in membrane potential in the dorsal root ganglion, cross-stimulation of type A fibres with type C fibres and the emergence of pain impulses. Pain impulses that arise can come from injured neurons or neurons that are still intact [11].

Sodium gates are the most important in the physiology of excitatory membranes, including neural membranes. One of the important findings for the occurrence of ectopic activation is the alteration of sodium gate expression in cell bodies and peripheral nerve endings after nerve injury. After peripheral nerve injury there is also a reorganization of the source and expression levels of various types of ion gates. Expression of some sodium gate subtypes is decreased, but others appear de novo or translocate to other parts of the neuron [11].

Neurotropin is known to be a crucial factor in the mechanism of alteration in sodium gate expression in peripheral nerve injury. It is known that in dorsal root ganglion culture, there will be an increase in the expression of some gate types and a decrease in the expression of others. In addition to the sodium gate, the calcium gate also plays a role in the development of hyperalgesia and allodynia after peripheral nerve injury [11].

Another peripheral mechanism that plays a role in the formation of neuropathic pain is the presence of collateral sprouting of severed nerves leading to the skin. The coupling mechanism between the sympathetic nervous system and the sensory nervous system plays a role in maintaining pain after nerve injury [11]. The increase in bradykinin, an inflammatory mediator, after nerve injury is considered to provide a bradykinin antagonist in the treatment of neuropathic pain [11].

Peripheral nerve injury causes neuropathic pain through gene regulation in the dorsal root ganglion and neuroinflammation in the spinal cord. In primary sensory neurons, MBD1 epigenetically suppresses the expression of opioid receptors and potassium channels subtype Kv1.2 (encoded by Kcna2). Kcna2 expression was also suppressed by long noncoding RNA (lncRNA). G9a activity in dorsal root ganglion neurons is increased after nerve injury, resulting in an epigenetic decrease in more than 40 potassium channel subtypes. The miR-183 cluster regulates the 2δ-1 and 2δ-2 subunits of voltage-gated calcium channel, the molecular target of gabapentin. In chronic pain, microglia activation in the spinal cord occurs where nerve injury and joint injury induce upregulation of MMP-9 and CSF-1 in dorsal root ganglion neurons, which then undergo axonal migration to the dorsal horn of the spinal cord. Upon release, MMP-9 and CSF-1 induce microglia activation (e.g. p38 phosphorylation) and microgliosis (proliferation and morphological changes) in the ipsilateral spinal cord, leading to the development of chronic pain.

This process then triggers spinal cord neuroinflammation, which is part of the central sensitization process. Upon activation, microglia produce and release IL-1β, which induces central sensitization and chronic pain via two presynaptic and postsynaptic regulatory pathways, leading to an increase in EPSC and a decrease in IPSC. IL-1β also modulates the activation of microglia and astrocytes in the spinal cord. Slow but persistent production of MMP-2 in astrocytes contributes to late-phase neuropathic pain. Both MMP-9 and MMP-2 are involved in regulating IL-1β cleavage and activation. Inhibition of MMP-9 and MMP-2 by TIMP-1 and TIMP-2 inhibits neuropathic pain [12].

### 2.2 Central mechanism

Central sensitization is a sequence of events in the dorsal horn that begins with the release of transmitters from nociceptors, changes in synaptic receptor density, changes in excitatory thresholds, all of which dramatically increase pain transmission. Central sensitization can also occur due to sensitization of nociceptors due to inflammation and spontaneous ectopic activity after nerve injury [13]. Central sensitization facilitates and enhances synaptic transfer from nociceptors to dorsal horn neurons. Initially, this process is triggered by nociceptor input to the spinal cord (activity dependent), then there is a molecular change in neurons (transcription dependent) [13].

The process of central sensitization begins with the activation of intracellular kinases, stimulates phosphorylation of ion gates and receptors, and changes in neuronal phenotype. Central and peripheral sensitization is a form of nervous system plasticity, where there is a change in function in response to changes in input (tissue damage). In a state of massive sensory flow due to severe tissue damage, within a few seconds the neurons in the spinal cord will become hyperresponsive. This reaction causes pain due to nonnoxious stimuli (eg pain due to light touch) and secondary hyperalgesia (pain in healthy tissue around damaged tissue) [13].

One of the receptors that plays a major role in these changes is the NMDA receptor. During the process of central sensitization, these receptors are phosphorylated and increase their sensitivity to glutamate. The excess response to glutamate is characterized by loss of magnesium ion blockade and a longer ion gate opening. Membrane excitability can be activated either by inputs that are below the threshold (subthreshold) or by excess responses to inputs above the threshold (suprathreshold). This phenomenon causes pain from a stimulus that is below the threshold (allodynia) and an exaggerated pain response to minimal stimulation (hyperalgesia), as well as an expansion of the sensitivity of the uninjured area (secondary hyperalgesia) [13].

Anatomical reorganization of the spinal cord after nerve injury is a change in the termination of nerve endings in the dorsal horn. The large myelinated Aβ nerve endings normally terminate in laminae III and IV but after injury they terminate more superficially in laminae II. The consequence of this reorganization is that there is additional input in the form of low threshold sensory neurons for second-order nerves [11].

Under normal conditions of pain stimulus, the central endings of Aδ and C nerve fibres release excitatory amino acids (EAA) such as glutamate and aspartate, neurotrophins (BDNF) and peptides (SP, neurokinin A, CGRP). BDNF will activate the tyrosine kinase B receptor while SP and Neurokinin A cause dorsal horn desensitization through their interaction with neurokinin 1 and 2 receptors.

## 3 MIR30C–5P

MicroRNAs are small non-coding RNAs with a length of about 19–25 nucleotides that play a role in embryonic growth, stem/progenitor cell differentiation. miRNAs also play an important role in regulating inflammation, apoptosis and oncogenesis processes. Sequence characteristics that can be inserted into a miRNA consist of 22 nucleotides, made from RNA of special sizes that are in complementary DNA sets, are phylogenetic conservationists, have hairpins which are important regulators [[14], [15], [16]].

MiRNAs modulate gene expression at the post-transcriptional level. The mechanical action of microRNA functions as an antisense regulator in the target gene by binding to the 3′ untranslated region (UTR) of mRNA which will inhibit gene expression. miR30c-5p is a family of microRNA30, namely miR30a, 30 b, 30c, 30 d and 30e. Studies of neuropathic pain in mice have found that microRNA-30c-5p has the potential as both a causative agent and a therapeutic target. Expression of microRNA-30c-5p is known to be increased in patients with pain due to diabetic neuropathy. Research on hepatocellular tumours and glioblastoma multiforme found that microRNA-30c is an inhibitor that inhibits tumour progression through inhibition of the proliferation and invasiveness of tumour cells and increases the sensitivity of tumour cells to chemotherapy. In patients with myocardial infarction reperfusion there is an increase in the expression of rno-miR-30c-5p which activates the NF-κB pathway and will trigger inflammation and increase apoptosis, so efforts to reduce the expression of rno-miR-30c-5p can be an alternative treatment for myocardial infarction [15,[17], [18], [19]].

## 4 TGF-β

TGF-β is one of the anti-inflammatory cytokines that can suppress glia activity in the spinal cord and suppress inflammation, and is a strong inhibitor of neuropathic pain. In response to nerve cell damage, the body releases pro-inflammatory cytokines such as IL1-β, TNF, IL-6, IL-15, IL-17, IL-18, IFN-ϒ which can increase pain while anti-inflammatory cytokines, such as IL- 4, IL-10 and TGF-β cause analgesics [19].

TGF-β inhibits lymphocyte proliferation and cytokine production, affects T-cell differentiation and increases the formation of immunosuppressive regulatory T-cells. In peripheral nerve injury TGF-β reduces the formation of pain and pre-existing pain through inhibition of activation of microglia and astrocytes, decreases CCL-2 expression and decreases BSCB integrity which will prevent further infiltration of immune cells in the dorsal horn of the spinal cord [19,20].

In chronic pain conditions, both humans and animals, downregulation of TGF-β results in reduced inhibition of neuroinflammation and microglia activation. Inhibition of neuropathic pain by TGF-β also occurs via gene transcription and neuromodulation pathways in the dorsal root ganglion. TGF-β rapidly in minutes activates TGF-β receptors on neurons which will normalize dorsal root ganglion hyperexcitability conditions due to nerve injury, and synaptic plasticity in the spinal cord. TGF-β is the target of miR-30c-5p, which is usually elevated in the spinal cord, dorsal root ganglion, cerebrospinal fluid and plasma after sciatic nerve injury in mice. TGF-β is downregulated by mir-30c-5p and upregulated by the inhibitor mir-30c-5p. This process is also thought to run in both directions through the opioid system [12].

## 5 VEGF

Vascular Endothelial Growth Factor (VEGF) is an endothelial growth factor that is able to stimulate the formation of blood vessels (angiogenesis). VEGF, or also called VEGF-A, is the oldest member of a growth factor consisting of VEGF, VEGF-B, VEGF-C, VEGF-D, VEGF-E and Placental Growth Factor (PlGF) which will bind to the receptor tyrosine kinase on the surface cells named VEGFR-1, VEGFR-2, VEGFR-3 and non-tyrosine kinase receptors from the neurophilin family, namely NRP-1, NRP-2, which will function as VEGFR co-receptors [21].

In the development of the nervous system, VEGF has two functions, namely regulating the formation of blood vessels and as a guide for nerve cell migration and axonal pathfinding, where the second function is not related to effects on blood vessels. The extracellular matrix bound to VEGF regulates the migration of cerebellar granule cells to their final destination via VEGFR-2 signalling within their granules, as well as the migration of facia-branchial motoneurons in the hindbrain regulated via the co-receptor NRP-1 [21].

In a healthy brain, VEGF regulates microvascular density, controls vascular permeability and maintains the endothelial fenestration process in the choroid plexus, stimulates proliferation and increases neural stem cell (NSC) neurogenesis. Meanwhile, in conditions of nervous system disease, VEGF has an effect on various types of nerve cells. VEGF acts as a guardian of diseased neurons through neuroprotective effects, stimulates neurogenesis and differentiation of nerve cells, stimulates axon elongation and branching and increases synaptic plasticity [21].

At low levels, VEGF is needed for endothelial cells to survive and maintain the integrity of the BBB, which protects the brain against damaging substances. On the other hand, at high levels, VEGF will damage the BBB, causing neurological disease through several mechanisms, such as the release of neurotoxic proteins, which will produce reactive oxygen species and inflammation, which in turn triggers an immune response. In addition, high levels of VEGF will trigger the formation of fragile blood vessels and bleeding [21].

In experimental animal neuropathic pain, it is suspected that VEGF plays a role through binding to VEGFR1 and VEGFR2, whose levels increase in conditions of hyperalgesia and neuropathic pain. VEGF may increase pain via P2X32/3 receptors in dorsal root ganglia, because administration of anti-VEGF antibodies reduces hyperalgesia by inhibiting P2X32/3 receptor activation and inhibiting primary afferent transmission mediated by VEGFR2 receptors and P2X32/3 receptors. Administration of Anti-rVEGF reduces the expression of VEGFR2 and P2X32/3 so that neuropathic pain can be reduced. Suppression of VEGF expression using VEGF-A164siRNA led to a prolongation of the antiallodynic effect. Increased expression of VEGF-VEGFR1 causes neuropathic pain and also increases BBB permeability. It is known that VEGFR1 is expressed on endothelial cells of the spinal cord, whereas VEGFR2 is expressed on astrocytes. VEGF expression is an adaptation process to ischemia caused by compression in the cauda equina. VEGF itself has a role in stimulating axon elongation and increasing the survival of dorsal root ganglia neurons [[22], [23], [24]].

## 6 Lumbal spine stenosis

Lumbar Spinal Stenosis (LSS) is a clinical syndrome caused by narrowing of the spinal canal with disturbances in the structure of the nerves that surround the bones and soft tissues. Clinical symptoms are variable but appear as a result of neurovascular mechanisms, excitation of nerve roots, or mechanical compression of the spinal canal [1].

Degenerative LSS is a progressive disease involving all segments of the lumbar vertebra. The relative instability initiated by degeneration of the intervertebral disc causes hypermobility of the vertebral segments, resulting in increased pressure on the posterior facet joints, followed by narrowing of the intervertebral disc space, increased angle of extension, and hypertrophy of the facet joints, particularly the superior articular hypertrophy process. The articular hypertrophic process causes local joint stiffness (ankylosis). In addition, calcification or thickening of the yellow ligament is an important mechanism in stenosis. Joint tropism can also be a factor causing degenerative Lumbar Spinal Stenosis. This causes spinal canal stenosis and compression of nerve structures, which can cause intermittent neurogenic claudication due to congestion of epidural venous blood flow and increased vascular pressure.

In LSS, when the nerve roots are under increased pressure in the spinal canal, nerve ischemia and nerve conduction disturbances can occur. Ischemia can occur due to venous occlusion and contribute to LSS involving two or more segments. If the nerve roots are damaged, both peripheral and central sensitization can occur, which can lead to chronic pain [25].

### 6.1 Classification

Based on the aetiology, LSS is classified into primary (congenital) and secondary stenosis. Primary (congenital) stenosis is defined as bony dysplasia leading to narrowing of the spinal canal, with further division into idiopathic and achondroplasty congenital stenosis. Secondary stenosis can be degenerative caused by metabolic diseases (such as Paget's disease), tumours, infections, osteoarthritic changes or instability with or without spondylolisthesis [[26], [27], [28]]. Based on the location, LSS is divided into central zone stenosis, lateral recess, foraminal and extraforaminal [26].

The combination of the disc-osteophyte complex and ligamentum flavum hypertrophy results in central stenosis. Hypertrophy of the facet joints with the development of osteophytes causes lateral recess stenosis. Foraminal stenosis can result from a substantial loss of disc height, the appearance of a foraminal disc or osteophytes, or there is angulation in degenerative scoliosis, whereas extraforaminal stenosis is caused by herniation of the lateral disc [26].

There are several parameters used to assess the severity of stenosis on imaging radiology, but the classifications commonly used are mild, moderate, and severe stenosis. Mild stenosis was defined as a decrease of <1/3 in the space available for neural elements, moderate stenosis was defined as a decrease of 1/3–2/3 in the space available for neural elements, whereas severe stenosis was defined as a decrease of >2/3 in the space available for neural elements. Another classification was proposed by Schizas et al. that assessed the severity of stenosis based on the morphology of the dural sac on MRI [25,26,29].

An easier classification was proposed by Lee et al. that assessed the severity of stenosis based on the degree of separation of the cauda equina on MRI [25].

1. Grade 0 (no stenosis or mild stenosis)
There was no central lumbar canal stenosis and there was no obliteration of the anterior cerebrospinal fluid space.
2. Grade 1 (mild stenosis)
There is a mild central lumbar canal stenosis which refers to a mild obliteration of the anterior cerebrospinal fluid space and all the cauda equina are clearly separated from each other.
3. Grade 2 (moderate stenosis)
There is moderate lumbar canal central stenosis which refers to moderate obliteration of the anterior cerebrospinal fluid space and multiple cauda equina aggregations that cannot be identified visually.
4. Grade 3 (severe stenosis)
There is severe lumbar canal central stenosis referring to severe obliteration of the anterior cerebrospinal space, signs of dural sac compression are present, and the entire cauda equina appears as a bundle.

LSS is clinically classified according to grades 1, 2 and 3. Grade 1 (neurogenic intermittent claudication) is characterized by reduced walking distance caused by pain and brief intermittent sensory deficits that are normal at rest but may worsen with walking. Grade 2 (intermittent paresis) refers to the presence of persistent sensitivity deficits, loss of reflexes, and intermittent paresis [28]. Grade 3 is achieved if there is persistent, progressive paresis accompanied by partial regression of pain [28].

### 6.2 Pathophysiology

Neuropathic pain can be caused by nociceptive lesions in the degenerating disc. In addition, it can also be caused by mechanical compression of the nerve roots or by the effects of inflammatory mediators arising from degenerative discs resulting in inflammation and damage to the nerve roots [30].

### 6.3 Clinical manifestation and diagnosis

The diagnosis of LSS can be made based on clinical presentation, physical examination and investigations. Clinical symptoms that usually appear in patients with LSS include unilateral or bilateral back pain accompanied by pain in the legs that develops and lasts for several months to years. The back pain is localized to the lumbar spine and may radiate to the gluteal region, groin and legs, often showing a pseudo radicular pattern. In the case of lateral recess stenosis or foraminal stenosis symptoms may occur in the form of radiculopathy. Neurogenic claudication is the most specific symptom of LSS although it is almost always accompanied by further symptoms [28].

Clinically, neurogenic claudication can be distinguished from intermittent vascular claudication on the basis of symptoms of pain regression after flexion (de-lordosis) of the spine (e.g. while cycling). In contrast to vascular claudication, pain sensation in patients with LSS does not subside on standing and has a relative proportion of lower back pain (as an indicator of pathology such as concurrent spinal instability or facet joint arthrosis) with a component of leg pain. In addition, it is known that about 20% of patients with LSS exhibit depressive symptoms and 25% feel dissatisfied with their lives before surgery [28].

Neuropathic pain in the case of LSS has the same quality of pain as lumbar radiculopathy, namely pain that radiates from the back through the buttocks to the lower extremities. In LSS Movements such as walking and descending stairs (lumbar extension) can exacerbate pain, while movements such as sitting will relieve pain [31].

Neuropathic pain caused by LSS can be sharp pain and numbness. This can be seen through the LANSS (Leads Assessment of Neuropathic Symptoms and Signs) scores. The characteristics of neuropathic pain include pain when light touches such as when wearing clothes, pain such as an electric shock that occurs suddenly, hot pain, allodynia and a positive Pin Prick threshold (PPT). Based on research conducted by Park et al., 2015 it was found that 1/3 of patients with LSS met the characteristics of neuropathic pain based on the LANSS score [1].

Furthermore, physical examination in the form of a lasegue test (passive leg stretching test) is often found with negative results in LSS patients accompanied by a feeling of heaviness in the legs. On investigation, a plain lumbosacral x-ray can be used as a routine assessment for patients with back pain. When performed in the lateral and oblique AP positions, the lumbosacral junction cone will appear, and the spine is in flexion and extension. This is done to obtain information on segment instability and deformity. Radiographic findings that lead to suspicion of degenerative lumbar stenosis are degenerative spondylolisthesis and degenerative scoliosis [28,[32], [33], [34]].

A CT (Computed Topography) scan can also be done to evaluate the bone, especially in the lateral recess. In addition, CT scans can also provide visualization of facet joint abnormalities, lateral disc abnormalities that lead to suspicion of lumbar stenosis, and can differentiate stenosis secondary to fracture [28,[32], [33], [34]].

Magnetic resonance imaging (MRI) has become the gold standard for the diagnosis of lumbar stenosis and as a medium for planning surgery. The advantage of MRI is that it can access the number of affected segments and can evaluate if there is a tumour or infection. In addition, MRI can also differentiate between central and lateral stenosis, seeing capsular thickening, facet joint abnormalities, osteophytes, disc herniation, and the presence or absence of epidural fat. MRI with a sagittal view can show the spine to look for the possibility of tumours that metastasize to the spine. MRI with a combination of axial and sagittal sections can also evaluate the central canal and neural foramen [28,[32], [33], [34]].

### 6.4 Treatment

Management in cases of Lumbar Spinal Stenosis can be divided into non-surgical management and surgical management. Non-surgical management can be performed in patients with mild to moderate symptoms. Non-surgical management can be in the form of medication, physiotherapy, spinal injection, lifestyle modification, and rehabilitation. Other treatments include aerobic orthosis, thermal therapy, ultrasound, massage, electrical simulation, and traction therapy. While surgical management can be decompressed with laminectomy [25,29].

## 7 MLC901

MLC901 (NeuroAiD II) is a combination of 9 herbal components whose efficacy and safety have been extensively studied preclinically and clinically. In in vitro and in vivo animal experiments MLC901 has neuroprotective and neuro-proliferative properties by facilitating the restoration of neuronal circuits through antioxidant effects, promotion of cell proliferation and stimulation of axon and dendrite neuronal circuits after brain injury. In general, there are 3 positive effects of MLC901 namely neurogenesis, neuro-restoration and neuroprotection with the latter effect being weaker than the others. Ischemic injured mice given MLC901 showed increased survival, neurological recovery, decreased neurodegeneration and improved cognitive function [[34], [35], [36], [37], [38]].

Administration of MLC901 can prevent excitotoxicity by inhibiting excessive Ca2+ influx, activating KATP channels that induce hyperpolarization conditions and preventing the release of excitotoxic glutamate, especially in neurons suffering from energy deficiency. KATP channel activation is also due to the effect of MLC901 which inhibits glibenclamide, a specific inhibitor of KATP [39,40].

MLC901 causes decreased apoptosis by lowering levels of the Bax protein, which is a potent proapoptotic molecule, a member of the Bcl-2 family that triggers terminal caspase activation [36,39].

In vitro experiments on cortical cell culture have shown that MLC901 helps develop axonal and dendritic arborization and dense neuritic tissue with elongation and increased number of branching. This increase in density and branching has a strong correlation with increased synaptogenesis [39].

One possible mechanism of the previously described effect of MLC901 includes its ability to stimulate the secretion of BDNF which is an important growth factor that regulates neuronal survival and brain plasticity. In vitro data showed that 6 weeks (6 mg/ml) of MLC901 treatment increased BDNF expression in cortical neurons [39,41].

Under ischemic conditions, the effect of MLC901 on increasing angiogenesis appears to be by modulating the expression of angiogenesis-related factors such as Hif1α, EPO, VEGF and Ang1/Ang2. These factors are known to not only mediate endothelial cell proliferation, but also regulate vascular differentiation, regression, and permeability [5].

MLC901 can serve as both treatment and prevention. Acute intraperitoneal administration (1 g/rat in a 500 l bolus) induced high survival rates and drastically reduced cerebral infarction with a reduction in stroke volume of about 50% compared to control mice at 30 h after ischemia. MLC901 is also effective in prevention, because initial treatment of MLC901 for 6 weeks given in drinking water (6 mg/ml) prior to ischemia induction led to a marked reduction in mortality of treated animals and a reduction in their brain infarction [39].

## 8 Peripheral nervous disorders

### 8.1 Causes of peripheral nerve disorders

Causes of peripheral nerve lesions can be divided into mechanical trauma, crush injury, laceration injury, pulling trauma & cold injury [42].

### 8.2 Classification of peripheral nerve lesions

Various classifications of peripheral nerve lesions have been made, but a good classification should be based on:

1. Damage to nerve fibre components
2. The presence of impaired function of nerve fibres
3. Ability to “heal” spontaneously

Based on this, in 1943 Seddon classified peripheral nerve lesions into 3, namely:

1. Neuropraxia: short-term loss of peripheral nerve function due to conduction disturbances, no damage to the nervous system and can return to normal in no more than 3 weeks
2. Axonotmesis: There is damage to the axon structure but the endoneurium tissue is still intact, can heal spontaneously, but Wallerian degeneration can also occur on the distal side of the lesion
3. Neurotmesis: There is damage to axons, myelin and tissue components surrounding the perineurium or epineurium. Spontaneous healing is not possible, often at the site of the lesion.

In 1951 Sunderland divided peripheral nerve lesions into 5 grades, namely [43]:

1. Grade I = neuropraxia
2. Grade II = axonotmesis
3. Grade III = damage to the axon and endoneurium but the perineurium is still intact, so it cannot heal spontaneously, intraneural fibrotic tissue formation occurs.
4. Grade IV = fasciculus damage (axon, endoneurium and perineurium but epineurium remains intact.
5. Grade V = complete damage, resulting in the formation of fibrotic tissue between the lesions

In 1988 Makinnon and Dellon introduced Sunderland's grade VI classification with more complex peripheral nerve damage [43].

## 9 Animal lesion models

### 9.1 Peripheral nerve lesion model

Neuropathic pain can occur after peripheral nerve injury or central nerve injury, also caused by ischemia, metabolic disorders or the presence of toxins. Models of neuropathic pain in experimental animals attempt to approximate this pattern of causes. The model of neuropathic pain due to spinal cord injury is obtained through direct damage in the form of contusions, surgical lesions, laser radiation to cause ischemia, the use of neurotransmitters that cause ecotoxicity. This spinal cord model causes mechanical and thermal hyperalgesia which can be seen by changing the behaviour of experimental animals through place preference tests [44].

Neuropathic pain models due to peripheral nerve disorders can be in the form of spinal nerve ligase/cutting, sciatic nerve ligase/lesion, spinal nerve branch ligase (SNI/spared nerve injury), injection of inflammatory irritants (zymosan). Although behavioural tests can't differentiate between the types of injury we're given, they can provide a decreased withdrawal threshold for hot and painful stimuli, as well as spontaneous movement of the injured extremity. Several disorders such as trauma, compression, infection, metabolic diseases, neurotoxins, tumours and others are responsible for the onset of neuropathic pain in humans. This neuropathic condition in humans has long been imitated in animals with peripheral neuropathic pain models, such as spinal nerve ligation, spared nerve injury, chronic constriction injury, partial nerve injury [45].

### 9.2 Chronic compression of the dorsal root ganglion

In this model the dorsal ganglion L4 and L5 are pressed using stainless steel rods. Chronic stress produces pain-related behaviors on the ipsilateral side, such as cutaneous allodynia, spontaneous pain that occurs 1 day after nerve injury and persists for more than 1 month. The effect of chronic compression of the dorsal root ganglion is thought to be related to increased excitability of nerve cells due to ganglion compression and sympathetic nervous system activity. In humans, disc protrusion or spinal stenosis can compress the dorsal root ganglion and cause low back pain and sciatica pain [44,45].

### 9.3 spinal nerve ligation

In this mononeuropathy trial, unilateral L5 and L6 spinal nerves distal to the dorsal root ganglion were subjected to strong ligation to produce hyperalgesia and allodynia within 24 h and no later than 4 months. In this model variant only the L5 is ligated. This model can be compared to the condition of dorsal root or plexus nerve injury in humans. Researchers can assess changes in axons including redistribution of sodium gates which is a condition for the emergence of neuropathic pain [44,45].

### 9.4 Partial nerve ligation

This method was used to mimic the spontaneous onset of pain in humans after partial nerve injury, by performing strong unilateral suturing and tying of nearly half of the sciatic nerve. Experimental animals will exhibit behaviour similar to chronic nerve constriction that appears within hours and lasts for more than 7 days. This method can also cause pain on the contralateral side, giving rise to bilateral pain symptoms. Sympathectomy and skin cooling can reduce the symptoms that appeared in this trial [44].

### 9.5 Spared nerve injury

Unilateral ligation of 33–50% of the sciatic nerve by separating the distal branch of the upper thigh results in partial nerve injury. This model is based on the separation and ligation of two of the three branches of the sciatic nerve: the common tibial and peroneal nerves are ligated while the sural nerves are left intact. The difference with some other neuropathy models is the presence of a limited degenerative area of the axon, making it possible to conduct behavioural experiments between the unaffected skin area and the denervated skin area [44,45].

### 9.6 Chronic constriction injury (CCI)

In this model of a very painful neuropathic lesion, the sciatic nerve is chronically constricted by means of a loose band at mid-thigh. This results in neuroinflammation associated with extensive deafferentation distal to the ligation. This model can be used to investigate pain-related behaviours including cold and mechanical allodynia, heat and chemical hyperalgesia. This model is similar to the clinical condition of chronic nerve compression in humans that can occur after lumbar disc protrusion or pinched nerves, heavy metal poisoning, anoxia and metabolic disorders [44,45].

### 9.7 Cuff neuropathy

Similar to CCI but by placing spherical polyethylene of the same diameter along the sciatica to reduce inter-investigator interpretation due to loose binding. In contrast to CCI, cuff neuropathy produces consistent changes in the fibres distal to the lesion. Changes that occur such as permanent decline in large myelinated axons, but only transient in small myelinated and unmyelinated axons. As the loss of large myelinated axons decreases the function of the cuff, it does not correlate with nocifensive behaviour, so it is suspected that there are other factors such as inflammation as a causative factor for neuropathy besides axon loss [44,45].

### 9.8 Axotomy

To induce a neuroma, the sciatic nerve is firmly ligated at mid-thigh using silk thread and then excised. The distal end of the neural tube is left exposed. Approximately 5 mm distal to the stump tip was excised to prevent regeneration. The nerve endings that make up the neuroma exhibit hyperexcitability and mechanical sensitization and are an interesting model of nerve injury [45].

### 9.9 Nerve destruction

In this model the sciatic nerve is opened at mid-thigh and is crushed using serrated haemostat forceps. Hyperalgesia and allodynia to touch and temperature appear within 3 weeks and last at least 52 weeks. Damaged nerves undergo Wallerian degeneration as well as undergo a regeneration process [45].

### 9.10 Spinal stenosis

Compression of the cauda equina is usually performed by applying compression through silicone placed on the dorsal part of the L4-L5 epidural. There are also those who regulate the degree of stenosis to avoid the occurrence of epidural fibrosis. Changes in behaviour appear on the first day until day 14. Cause behavioural disorders, intermittent neurogenic claudication. The circumferential model of spinal stenosis is still rarely used and can cause motor abnormalities, electrophysiological abnormalities of nerve fibres and demyelination which are part of the symptoms of neuropathic pain [44,46,47].

### 9.11 Conclusion

Neuropathic pain is defined as a pain that is initiated or caused by a primary lesion or dysfunction of the nervous system and may arise as a result of lesions or diseases affecting the somatosensory system. Peripheral nerve injury causes neuropathic pain through gene regulation in the dorsal root ganglion and neuroinflammation in the spinal cord. Neuropathic pain is a major problem whose pathogenesis is unknown and difficult to treat. Effective treatment of neuropathic pain usually uses multimodal therapy that takes a long time but causes major health problems. The narrowing of the spinal canal resembles an ischemic condition that can increase the expression of VEGF in the dorsal root ganglion and then result in shortened walking distance (intermittent claudication). The effect of VEGF is thought to be through binding to VEGFR1 and VEGFR2, whose levels are increased in conditions of hyperalgesia and neuropathic pain. Immune mechanisms play a role in the pathogenesis of neuropathic pain, through the balanced process of pro-inflammatory cytokines and anti-inflammatory cytokines, TGF-β, which are immunosuppressive.

MLC901 is a simplified traditional medicine formula from MLC601, which affects the nervous system through three main mechanisms, namely neuroprotection, neuro-regeneration and neuro-restoration. Elevated levels of MLC901 promote angiogenesis. The effect of MLC901 on miR30c-5p expression, TGF-β expression, VEGF receptor expression, degree of axon demyelination and changes in neuropathic pain behaviour in experimental animals experiencing neuropathic pain using the circumferential spinal stenosis method. In experimental animal models, trauma conditions are treated similar to those of nerve injury in humans. Among others: Compression of the dorsal root ganglion, Spinal Nerve Ligation, Partial Nerve Ligation, Spared Nerve Injury, Chronic Constriction Injury, Cuff neuropathy, Axotomy, Nerve Destruction, and Cauda Equina Compression. These findings may provide new targets for further scientific research on the molecular mechanisms of neuropathic pain and potential therapeutic interventions.

Need more research and study to know how effect some injury spinal nerve in animals model or humans between giving MLC901 and MLC601 contain medication.

## Ethical approval

This article is a review, hence no ethical approval is required for this type of study.

## Sources of funding

No funding or sponsorship.

## Author contribution

BAM, RHA, AAI, MH and AGB contributed to the concept and design, analysis and interpretation of data, literature search, data extraction and wrote the paper. BAM, RHA, AAI, MH and AGB supervised in different stages and contributed to the interpretation of data, revising the manuscript for important intellectual content and approval of the final manuscript. BAM, RHA, AAI, MH and AGB were involved in drafting and revising the manuscript and approved the final version.

## Registration of research studies

Name of the registry: Not applicable.

Unique Identifying number or registration ID: Not applicable.

Hyperlink to your specific registration (must be publicly accessible and will be checked): Not applicable.

## Guarantor

Rohadi Muhammad Rosyidi.

## Provenance and peer review

Not commissioned, externally peer reviewed.

## Declaration of competing interest

The authors declare that they have no conflict of interests.

## References

[1] Park S.Y., An H.S., Moon S.H., Lee H.M., Suh S.W., Chen D., Jeon J.H. (2015). Neuropathic pain components in patients with lumbar spinal stenosis. Yonsei Med. J..

[2] Hopley L dan, van Schalkwyk J. (2006). Pain physiology. http://www.anaesthetist.com/icu/pain/Findex.htm#index.htm.

[3] Song K.S., Cho J.H., Hong J.Y., Lee J.H., Kang H., Ham D.W., Ryu H.J. (2017). Neuropathic pain related with spinal disorders: a systematic review. Asian Spine Journal.

[4] Scholz J., Woolf C.J. (2007). The neuropathic pain triad: neurons, immune cells and glia. Nat. Neurosci..

[5] Gandin C., Widmann C., Lazdunski M., Heurteaux C. (2016). MLC901 favors angiogenesis and associated recovery after ischemic stroke mice. Cerebrovasc. Dis..

[6] Chen (2018). https://www.cell.com/neuron/pdf/S0896-6273(18)30844-4.pdf.

[7] Colloca L., Ludman T., Bouhassira D., Baron R., Dickenson A.H., Yarnitsky D., Freeman R., Truini A., Attal N., Finnerup N.B., Eccleston C., Kalso E., Bennett D.L., Dworkin R.H., Raja S.N. (2017). Neuropathic pain. Nat. Rev. Dis. Prim..

[8] Helme Robert (2006). Drug treatment of neuropathic pain. Aust. Prescr..

[9] Purba (2016).

[10] Vranken J.H. (2011). Neuropathic pain following spinal cord injury. Neuropathic Pain: Causes, Management, and Understanding.

[11] Bridges D., Thompson S.W.N., Rice A.S.C. (2001). Mechanisms of neuropathic pain. Br. J. Anaesth..

[12] Buchheit T., Huh Y., Maixner W., Cheng J., Ji R.R. (2020). Neuroimmune modulation of pain and regenerative pain medicine. J. Clin. Invest..

[13] Woolf C.J. (2004). Pain: moving from symptom control toward mechanism-specific pharmacologic management. Ann. Intern. Med..

[14] Winter J., Jung S., Keller S., Gregory R.I., Diederichs S. (2009). Many roads to maturity: MicroRNA biogenesis pathways and their regulation. Nat. Cell Biol..

[15] Chen J., Zhang M., Zhang S. (2020). Rno-microRNA-30c-5p promotes myocardial ischemia reperfusion injury in rats through activating NF-κB pathway and targeting SIRT1. BMC Cardiovasc. Disord..

[16] Meštrović T. (2018). https://www.news-medical.net/life-sciences/MicroRNA-Nomenclature.aspx.

[17] Hu S., Zhang J., Fang X., Guo G., Dai J., Sheng Z., Li D., Chen J., Zhang L., Liu C., Gao Y. (2021). Identification of microRNA hsa-miR-30c-5p as an inhibitory factor in the progression of hepatocellular carcinoma and investigation of its regulatory network via comprehensive analysis. Bioengineered.

[18] Liu S., Li X., Zhuang S. (2019). miR-30c impedes glioblastoma cell proliferation and migration by targeting SOX9. Oncology Research.

[19] Tramullas M., Francés R., De La Fuente R., Velategui S., Carcelén M., García R., Llorca J., Hurlé M.A. (2018). MicroRNA-30c-5p modulates neuropathic pain in rodents. Sci. Transl. Med..

[20] Austin P., Moalem-Taylor G., Toth C., Moulin D. (2011). Neuropathic Pain: Causes, Management and Understanding.

[21] Lange C., Storkebaum E., De Almodóvar C.R., Dewerchin M., Carmeliet P. (2016). Vascular endothelial growth factor: a neurovascular target in neurological diseases. Nat. Rev. Neurol..

[22] Kikuchi M.S.Æ.S. (2007). Spinal stenosis : assessment of motor function. VEGF expression and angiogenesis in an experimental model in the rat.

[23] Lee G.W., Son J.Y., Lee A.R., Ju J.S., Bae Y.C., Ahn D.K. (2019). Central VEGF-A pathway plays a key role in the development of trigeminal neuropathic pain in rats. Mol. Pain.

[24] Lin J., Li G., Den X., Xu C., Liu S., Gao Y., Liu H., Zhang J., Li X., Liang S. (2010). VEGF and its receptor-2 involved in neuropathic pain transmission mediated by P2X 2/3 receptor of primary sensory neurons. Brain Res. Bull..

[25] Lee B.H., Moon S.H., Suk K.S., Kim H.S., Yang J.H., Lee H.M. (2020). Lumbar spinal stenosis: pathophysiology andTreatment principle: a narrative review. Asian Spine Journal.

[26] Schroeder (2016).

[27] Sa (2014).

[28] Siebert (2009).

[29] Lurie J., Tomkins-Lane C. (2016).

[30] Baron R., Binder A., Attal N., Casale R., Dickenson A.H., Treede R. (2016). Neuropathic low back pain in clinical practice. Eur. J. Pain.

[31] Patel (2018). Endocannabinoid signaling collapse mediates stress-induced amygdalo-cortical strengthening. https://www.cell.com/neuron/pdf/S0896-6273(19)31090-6.pdf.

[32] Fortin & Wheeler (2004). https://www.painphysicianjournal.com/current/pdf?article=OTQ%3D&journal=18.

[33] Fraser et al. (2003). The modifier subunit of Drosophila glutamate-cysteine ligase regulates catalytic activity by covalent and noncovalent interactions and influences glutathione homeostasis in vivo. The modifier subunit of Drosophila glutamate-cysteine ligase regulates catalytic activity by covalent and noncovalent interactions and influences glutathione homeostasis in vivo. 10.1074/jbc.M308035200. 12954617

[34] Quintard H., Lorivel T., Gandin C., Lazdunski M., Heurteaux C. (2014). MLC901, a Traditional Chinese Medicine induces neuroprotective and neuroregenerative benefits after traumatic brain injury in rats. Neuroscience.

[35] Kumar R., Htwe O., Baharudin A., Ariffin M.H., Abdul Rhani S., Ibrahim K., Rustam A., Gan R. (2016). Spinal cord injury—assessing tolerability and use of combined rehabilitation and NeuroAiD (SATURN study): protocol of an exploratory study in assessing the safety and efficacy of NeuroAiD amongst people who sustain severe spinal cord injury. JMIR Research Protocols.

[36] Quintard H., Borsotto M., Veyssiere J., Gandin C., Labbal F., Widmann C., Lazdunski M., Heurteaux C. (2011). MLC901, a Traditional Chinese Medicine protects the brain against global ischemia. Neuropharmacology.

[37] Suwanwela N.C., Chen C.L.H., Lee C.F., Young S.H., Tay S.S., Umapathi T., Lao A.Y., Gan H.H., Baroque A.C., Navarro J.C., Chang H.M., Advincula J.M., Muengtaweepongsa S., Chan B.P.L., Chua C.L., Wijekoon N., De Silva H.A., Hiyadan J.H.B., Wong K.S.L., Ranawake U. (2018). Effect of combined treatment with MLC601 (NeuroAiD TM) and rehabilitation on post-stroke recovery: the CHIMES and CHIMES-E studies. Cerebrovasc. Dis..

[38] Theadom A., Barker-Collo S., Jones K.M., Parmar P., Bhattacharjee R., Feigin V.L. (2018). MLC901 (NeuroAiD IITM) for cognition after traumatic brain injury: a pilot randomized clinical trial. Eur. J. Neurol..

[39] Heurteaux (2015). Positive effects of the traditional Chinese medicine MLC901 in cognitive tasks.

[40] Ou Maati Moha (2012).

[41] Heurteaux C., Gandin C., Borsotto M., Widmann C., Brau F., Lhuillier M., Onteniente B., Lazdunski M. (2010). Neuroprotective and neuroproliferative activities of NeuroAid (MLC601, MLC901), a Chinese medicine, in vitro and in vivo. Neuropharmacology.

[42] Sharon I., Fishfeld C., Duong D.H., Talavera F., Wyler A.R. (2004). Acute nerve injury. http://www.emedicine.com/med/topic2908.htm.

[43] Grant G.A., Goodkin R., Kliot M. (1999). Evaluation and surgical management of peripheral nerve problems. Neurosurgery.

[44] Stemkowski P.L., Smith P.A. (2011). An overview of animal models for neuropathic pain. Neuropathic Pain: Causes, Management, and Understanding.

[45] Niederberger E., Kühlein H., Gisslinger G. (2009). Update on pathobiology of neuropathic pain. http://www.medscape.com/viewarticle/586220?src=mp&spon=26&uac=107126AJ.

[46] Cheung P.W.H., Hu Y., Cheung J.P.Y. (2019). Novel compression rat model for developmental spinal stenosis. J. Orthop. Res. : Official Publication of the Orthopaedic Research Society.

[47] Watanabe K. (2007).

